# Supplementary material for: The Major Components of Cerebrospinal Fluid Dictate the Characteristics of Inhibitors against Amyloid-Beta Aggregation
Source: Int J Mol Sci. 2023 Mar 22;24(6):5991. doi: 10.3390/ijms24065991 (PMC10059578; doi:10.3390/ijms24065991)
Supplement: Supplementary file 1 [file ijms-24-05991-s001.zip › Supplementary figures and table.pdf]

The Major Components of Cerebrospinal Fluid Dictate the Characteristics of Inhibitors against Amyloid-Beta Aggregation

Andrius Sakalauskas *, Mantas Ziaunys, Ruta Snieckute, Agne Janoniene, Dominykas Veiveris, Mantas Zvirblis, Virginija Dudutiene and Vytautas Smirnovas *

Institute of Biotechnology, Life Sciences Center, Vilnius University, LT-10257 Vilnius, Lithuania
* Correspondence: andrius.sakalauskas@gmc.vu.lt (A.S.); vytautas.smirnovas@bti.vu.lt (V.S.)

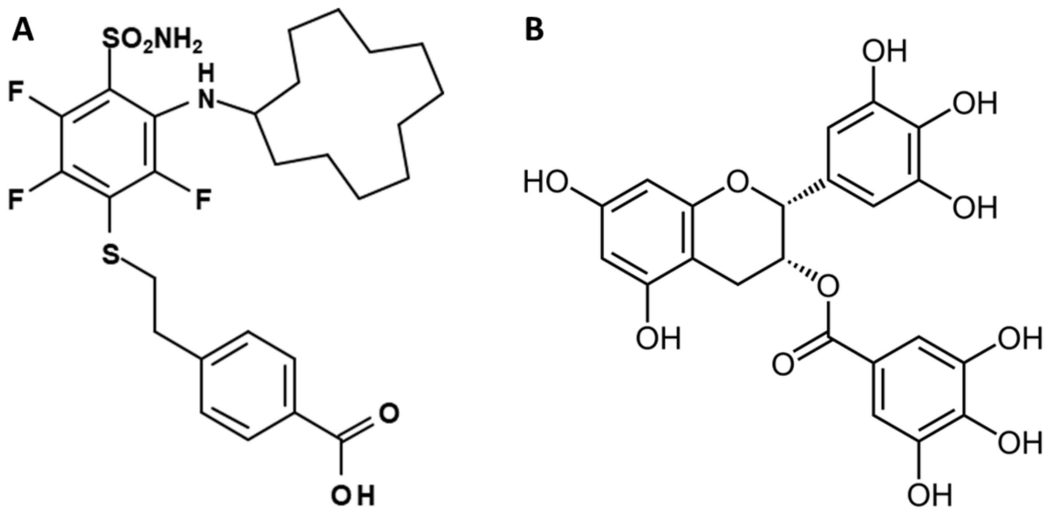

Figure S1. Chemical structure of VR16-09 (A) and EGCG (B).

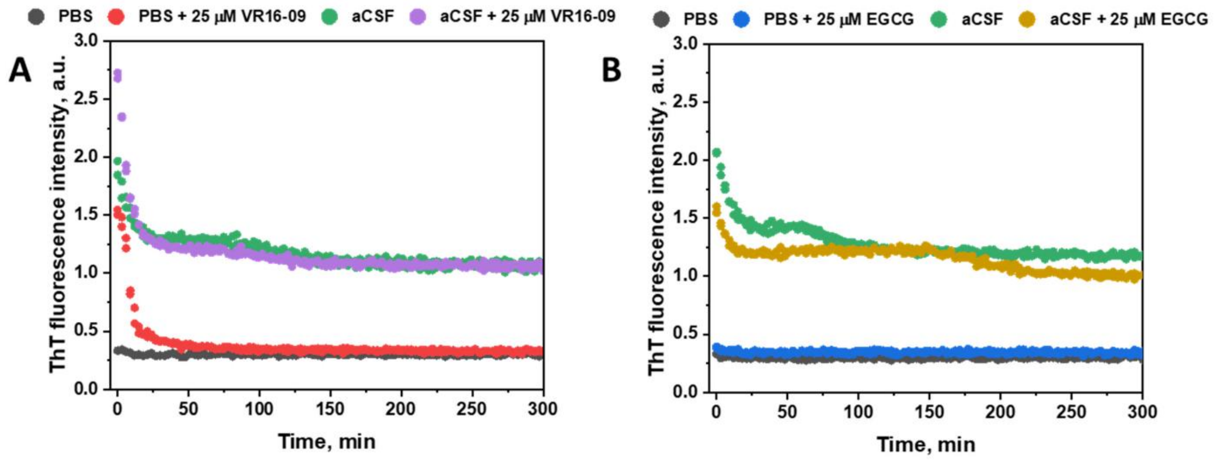

Figure S2. Fluorescence intensity curves of VR16-09 (A) and oxidized EGCG (B) in absence of $A\beta_{42}$. The curves are measured identically to the reaction of $A\beta_{42}$ aggregation in PBS and aCSF.

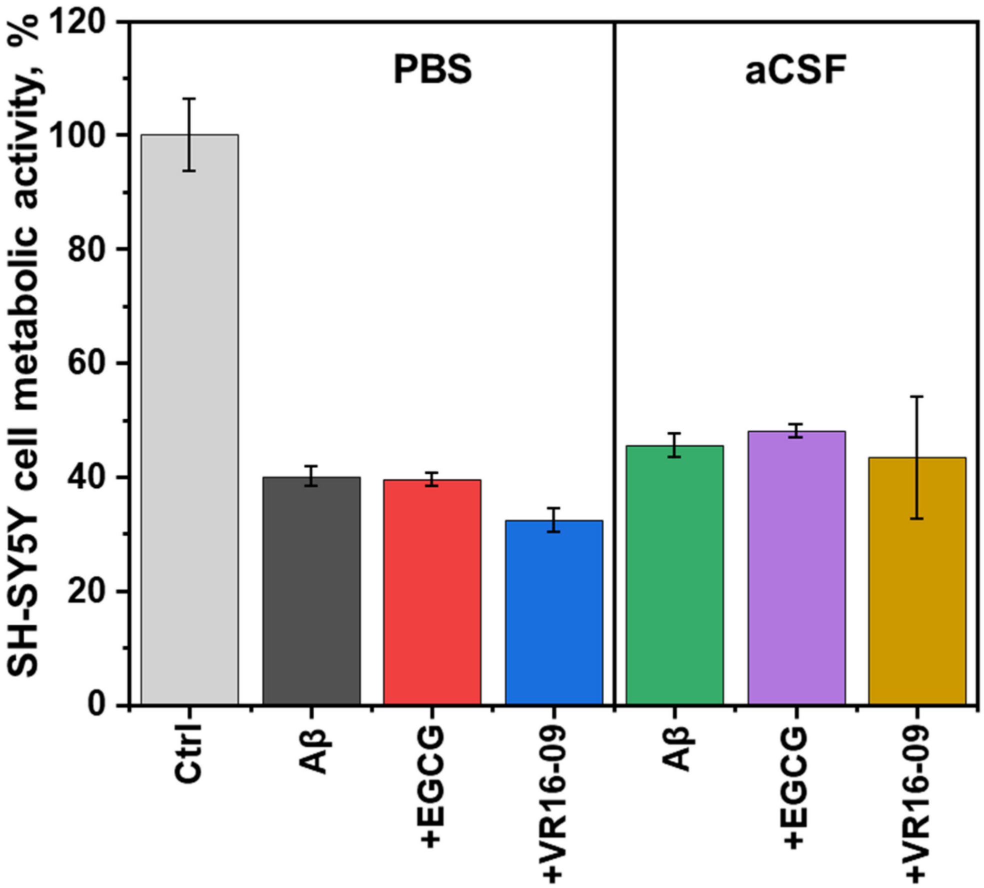

Figure S3. SH-SY5Y cell metabolic activity after incubating (48 hours) in medium containing $A\beta_{42}$ alone and with EGCG or VR16-09.

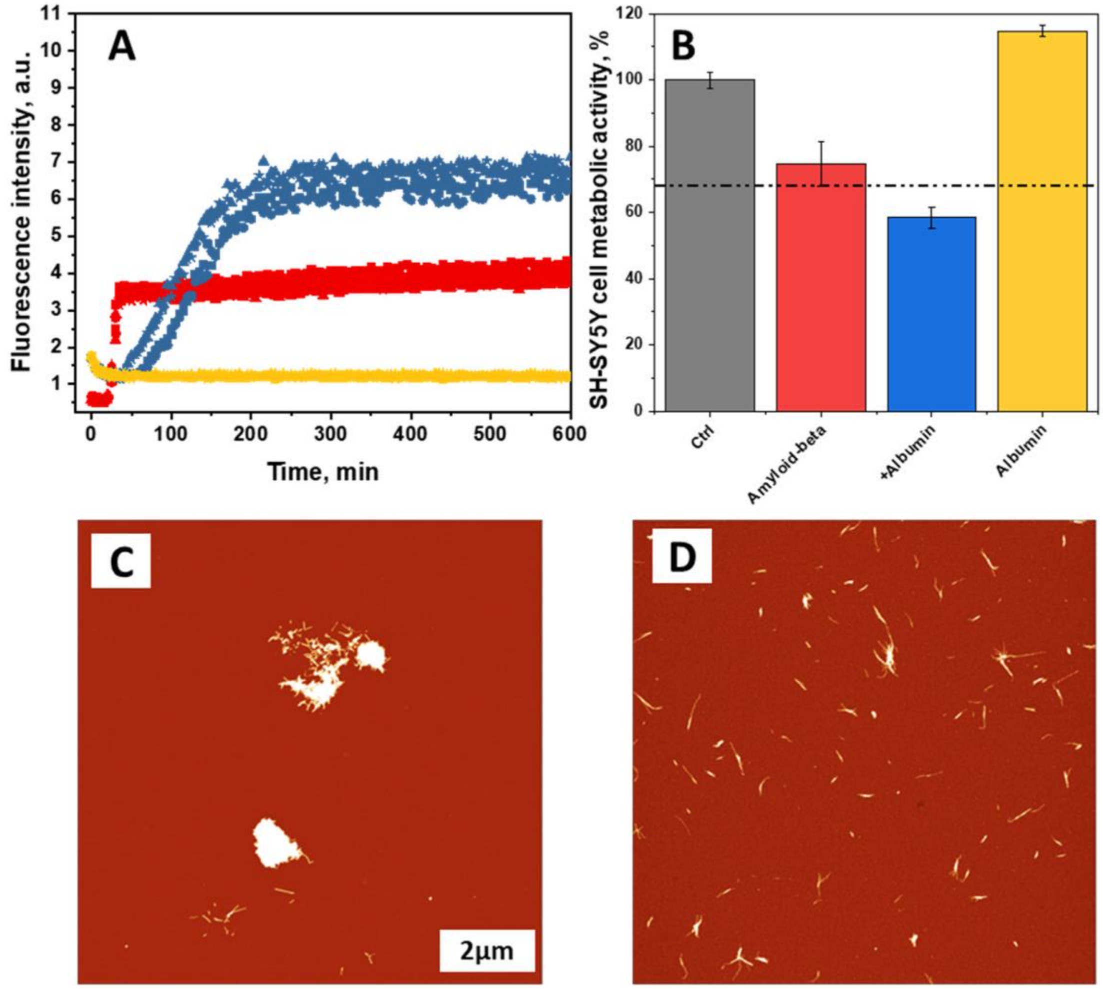

Figure S4. 2 μM of $A\beta_{42}$ aggregation (A) (Red), 2 μM $A\beta_{42}$ + 10 μM BSA (Blue) and 10 μM BSA (Yellow) kinetic curves. SH-SY5Y cell metabolic activity (B) after incubating (48 hours) with $A\beta_{42}$ and added albumin or albumin alone. Atomic force microscopy (AFM) images of 2 μM of $A\beta_{42}$ without (C) and with 10 μM of albumin (D).

Table S1. Halftime value data (in minutes) extracted from Boltzmann sigmoidal fits.

**Figure 1: X0**	**in PBS**				**in aCSF**			
Control	16.8	17.3	15.9	16.4	50.0	50.2	51.7	50.3
w/ EGCG	36.3	35.9	33.3	34.4	32.4	32.1	34.1	34.2
w/ VR16-09	19.0	18.9	16.8	18.1	115.6	117.8	108.5	106.7
**Figure 3: X0**	**w/o VR16-09**				**w/ VR16-09**			
Control	40.6	41.3	42.5		40.3	42.2	41.0	41.2
w/ Glucose	37.1	36.9	41.3	40.9	56.4	54.4	51.7	55.6
w/ $CaCl_2$	30.9	30.9	33.1	34.7	37.3	37.0	37.5	36.5
w/ $MgCl_2$	35.6	35.3	35.8	36.4	54.1	43.8	39.5	40.1
w/ Urea	36.4	37.6	36.5	38.7	48.9	48.2	43.9	47.2
w/ Glutamine	138.4	153.3	132.5	142.5	279.6	356.2	288.1	258.1
w/ Cholesterol	38.5	38.9	36.5	38.1	64.5	65.2	52.7	64.3
w/ Latcate	39.6	38.8	39.7	39.9	46.4	41.4	58.5	46.8
w/ HSA	78.9	78.6	68.5	78.2	53.8	56.5	50.9	49.2
aCSF	68.7	47.7	60.5	54.6	186.2	274.8	155.1	169.2
